# Early vasopressin application in shock

**DOI:** 10.1186/cc10375

**Published:** 2011-10-27

**Authors:** S Oliveira

**Affiliations:** 1 Albert Schweitzer and Hospital Bangu Hospital, Rio de Janeiro - RJ, Brazil

## Introduction

Vasopressin is frequently used to maintain blood pressure in refractory septic shock. We hypothesized that early infusion of vasopressin compared with norepinephrine would decrease the mortality rate and severity of septic status.

## Methods

In this randomized, double-blind study, we assigned patients who need vasopressors and randomized to receive norepinephrine (0.05 to 2.0 μg/kg/minute) or vasopressin (0.01 to 0.03 U/minute) with norepinephrine. Both groups had the vasoactive drug infusions titrated and tapered to maintain a mean blood pressure between 65 and 75 mmHg.

## Results

A total of 387 patients underwent randomization with 191 patients receiving vasopressin and 196 receiving norepinephrine. There was no significant heterogeneity between these two study groups. There was a significant difference between the vasopressin and norepinephrine groups in the mortality rate of 14 days (29.3% vs. 36.7%, respectively, *P *= 0.05) and 28 days (34% and 42.3%, respectively, *P *= 0.03); however, in 7-day mortality there were no significant differences in the overall rates (21.2% vs. 23.9%, respectively; *P *= 1.1). Also note a reduction in the incidence of single organ dysfunction (37.7% vs. 49.2%, respectively, *P *= 0.02) and multiple organ dysfunction using vasopressin and norepinephrine (17.8% vs. 26%, *P *= 0.05; *P *= 0.03). The length of stay in the ICU was 14 and 17 days (*P *= 0.29) and the time of hospitalization was 23 and 28 days (*P *= 0.11), respectively, in the vasopressin and norepinephrine groups.

## Conclusion

Early application of vasopressin reduced mortality rates in 14 and 28 days as compared with norepinephrine alone, and also a difference in incidence of organ dysfunction. This observed difference can be attributed to early restoration of tissue perfusion and vascular smooth muscle responsiveness that directly influenced patient survival.

